# Best Practices in the Prevention and Management of Intraoperative Intravenous Infiltration

**DOI:** 10.7759/cureus.90514

**Published:** 2025-08-19

**Authors:** Danielle A Hahn, Kyle Chambers, Gisele J Wakim

**Affiliations:** 1 Anesthesiology, University of Miami Miller School of Medicine, Jackson Memorial Hospital, Miami, USA

**Keywords:** intraoperative monitoring, iv infiltration, peripheral iv complications, quality improvement, surgical patient safety

## Abstract

Intravenous (IV) infiltration is a common complication of IV therapy, with complications that can range from mild swelling to damage to surrounding tissues and compartment syndrome. Intraoperative detection is particularly difficult when patients are anesthetized and unable to report symptoms or positioned in a way that may limit visibility of hands to appreciate early infiltration. We present the case of an 83-year-old male who developed hand swelling at the IV site during a right craniotomy for meningioma removal, initially unrecognized due to hand tucking for surgical positioning. This case highlights the need for standardized IV infiltration prevention and management strategies in the surgical setting, in which there may be restricted access or visualization of IV catheters. Quality improvement strategies in this setting would include approaches that promote catheter securement, regular monitoring of IV catheter sites, plans of action in the case of suspected infiltrated IVs, and interdisciplinary communication that may facilitate timely and organized interventions.

## Introduction

A frequent complication of IV therapy, infiltration is characterized by the unintended leakage of intravenous (IV) solutions into the surrounding tissue. Helm et al. found that infiltration is the most common form of peripheral IV catheter complication, with an occurrence rate ranging from 15.7% to 33.8% [1]. Some complications of infiltration, include swelling, extravasation (damage to surrounding tissues from release of vesicant fluids outside the intended vessel), and most severely, compartment syndrome (dangerous pressure buildup within a muscle compartment that can cut off blood flow to the muscles and nerves in the area), as described by Gibian et al. [2]. These complications may not only compromise patient safety but can also extend hospitalization and contribute to waste of hospital resources, as noted by Bahl et al. and Liu et al. [3,4]. Despite its clinical significance, there is limited literature discussing effective prevention and management strategies in the adult inpatient surgical setting. In the operating room, when patients are under anesthesia and unable to report symptoms of pain and swelling, or may have hands tucked for surgery, blocking visibility of hands to appreciate early infiltration, there may be delayed recognition and further risk of complications. Existing IV infiltration prevention measures in the operating room include careful site selection, securement with transparent dressings, scheduled IV checks, and the use of ultrasound-guided access for difficult veins. However, these strategies are not standardized or consistently applied as compared with prevention measures in general patient care outside of the operating room. By identifying and implementing evidence-based prevention and management strategies, there is an opportunity to improve patient perioperative care. This case report involves an 83-year-old male who developed hand swelling secondary to IV infiltration during craniotomy with delayed recognition. This case highlights the need for standardized IV infiltration prevention in surgical settings with restricted access to IV catheters, as well as standardized management protocols to maximize patient safety.

## Case presentation

**Figure 1:**
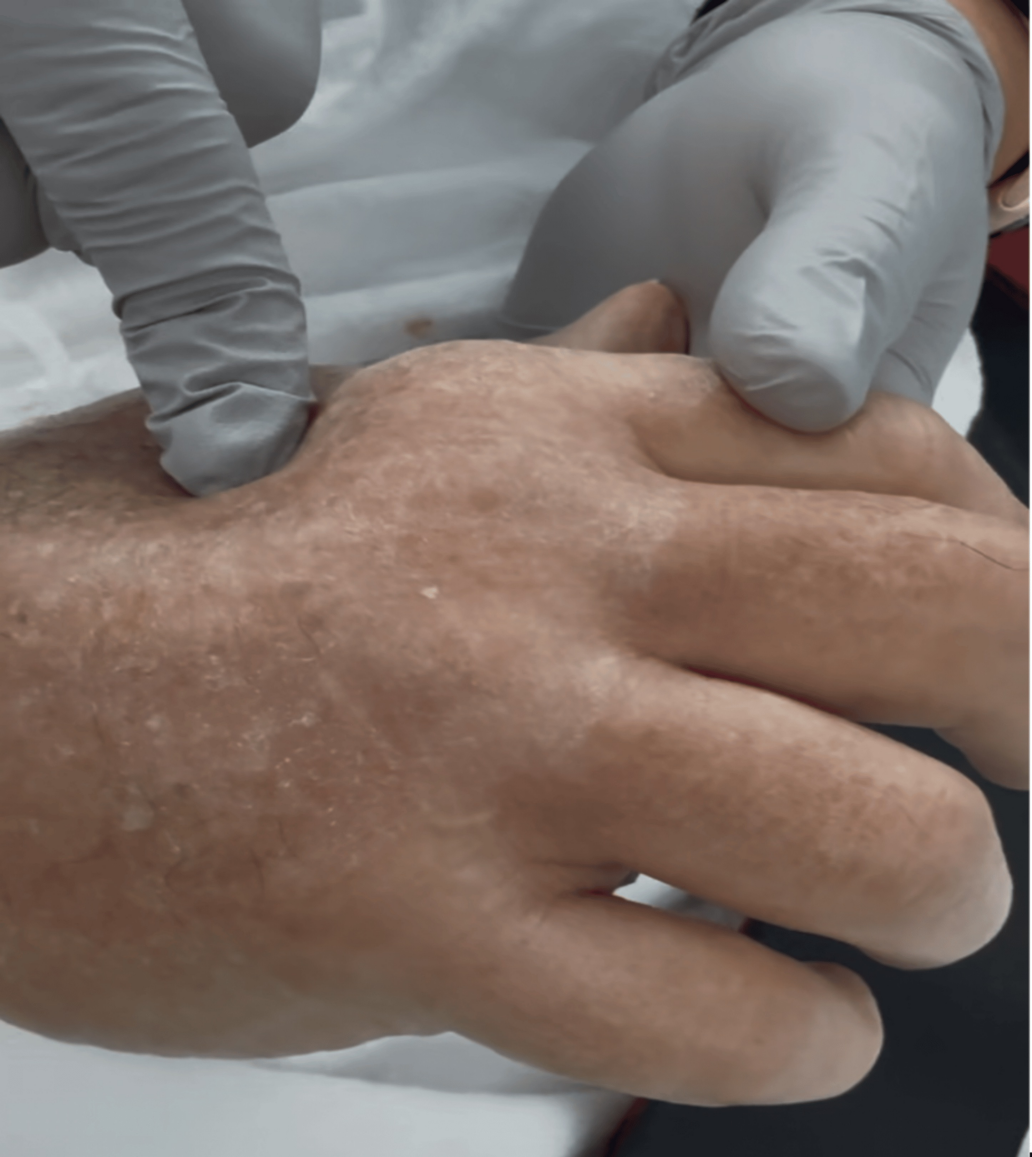
Postoperative image showing hand edema localized around the peripheral intravenous catheter insertion site.

An 83-year-old male presented for a right craniotomy for meningioma removal. Two 18-gauge IV catheters were placed, one in each hand. IV fluids were administered via the line in the right hand, while IV medications were infused through the line in the left hand. The patient was initially positioned supine for induction of general anesthesia with both arms tucked at the patient’s sides for improved surgical access and maintenance of a sterile field. Each arm was wrapped in a draw sheet, and the hands were placed in a supinated position. The anesthesia provider was cautious to avoid compression of the nerves in the elbow and wrist. The patient was then repositioned with his head rotated to the left for optimal surgical exposure. After final positioning, the IV catheters were checked for patency. The patient developed significant visible swelling at the dorsum of the right hand at the IV site, noticed by the anesthesia team towards the end of the surgery, as demonstrated in Figure 1. Recognition of the hand edema was delayed, due in part to the patient’s hands being tucked at his sides during surgery. 

**Figure 2:**
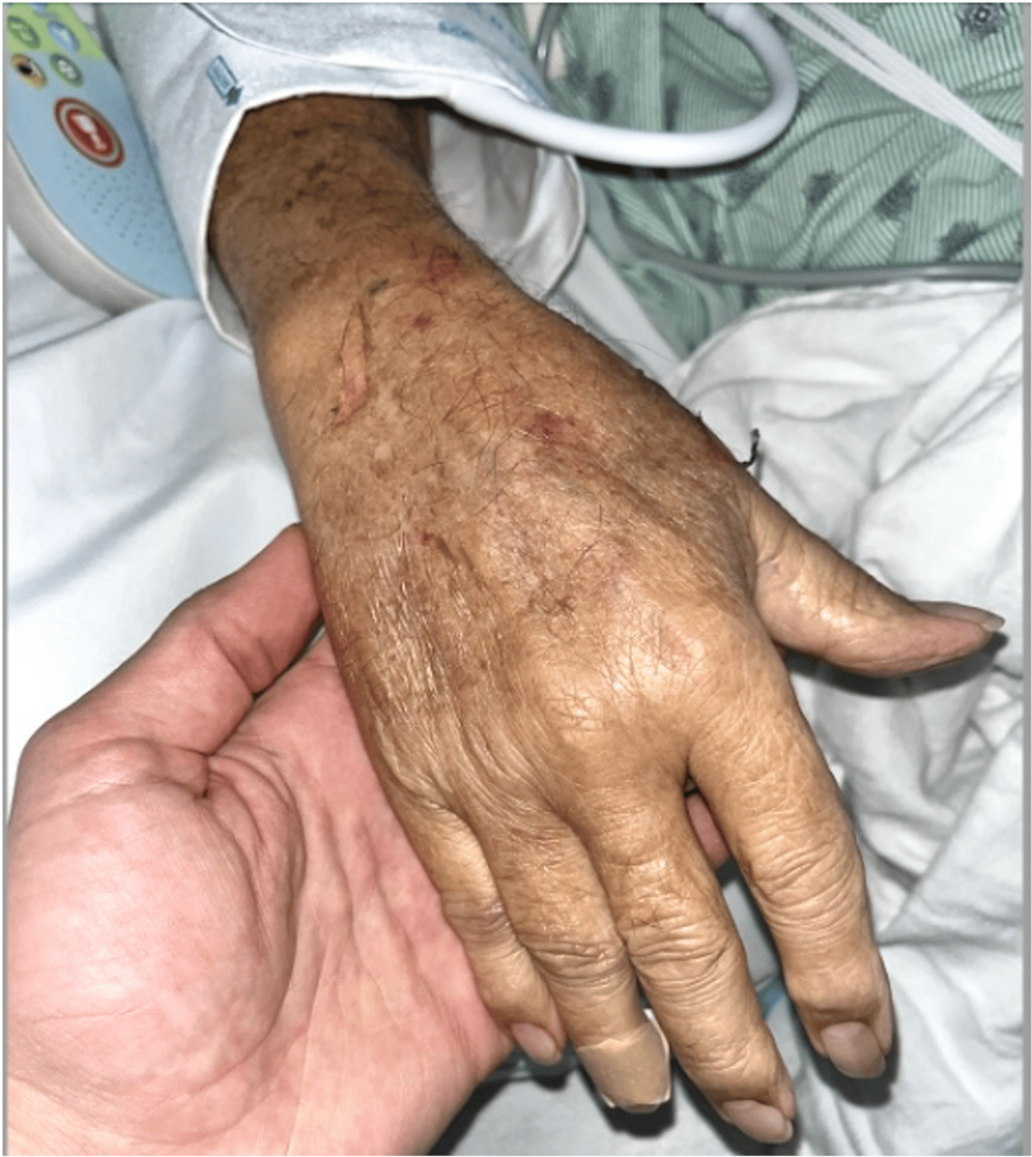
Post-intervention image of the dorsum of the hand showing reduced edema after intravenous catheter removal and limb elevation.

**Figure 3:**
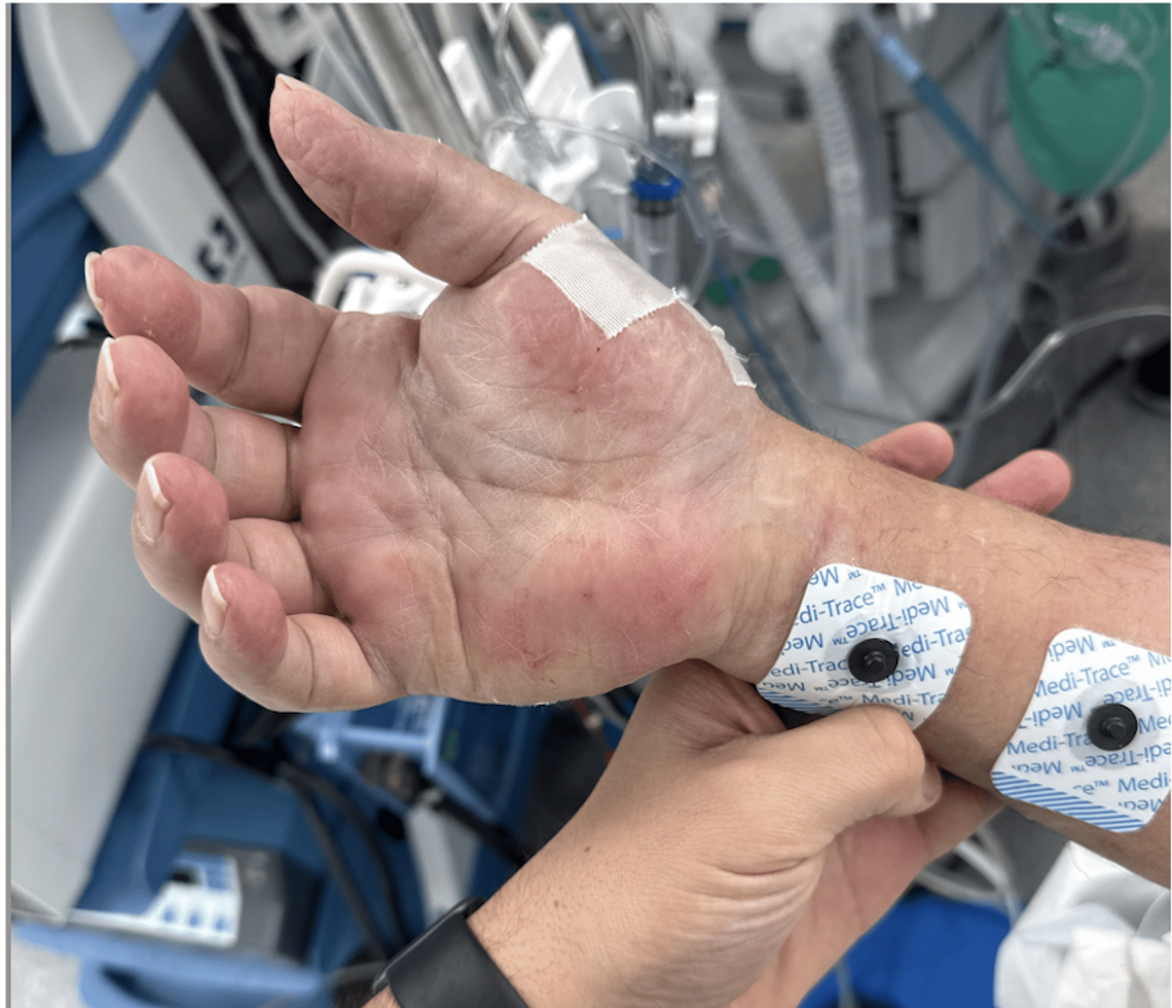
Post-intervention image of the palmar surface of the hand showing reduced edema and residual redness, and peeling after intravenous catheter removal and limb elevation.

While the anesthesia team noted some difficulty flushing the IV intraoperatively, the IV seemed to be functioning properly, and there were no other signs of IV-related complications. Postoperatively, an orthopedic hand specialist was consulted, and compartment syndrome was ruled out based on the absence of pain out of proportion to swelling, lack of firmness in the affected tendon compartments, absence of pallor, cyanosis, and excessive ecchymosis, as well as preservation of sensation, mobility, and pulse. Because there was marked improvement in symptoms after removal of the IV and with elevation of the right upper extremity (Figures 2, 3), it was determined by the anesthesia team that this complication was simply IV infiltration. Edema gradually decreased over 48 hours with near-complete resolution by postoperative day 3.

## Discussion

By definition, IV infiltration occurs when fluids leak exterior to the intended vein. In this case, the patient’s hand positioning during surgery likely contributed to the infiltration by impeding appropriate IV flow. The anesthesia team noted that while the IV was functional, it was difficult to flush, suggesting possible infiltration. Forceful flushing could have led to increased swelling or potentially the propagation of a clot. Difficult flushing should be an indication to utilize an alternate IV site. Quality improvement strategies for IV infiltration prevention and management in the surgical setting can be shown in Table 1 and expanded upon below [5-10].

**Table 1:** Evidence-based prevention and management strategies for intravenous (IV) infiltration in the operative setting.

Category	Rationale	Recommendations
1) Catheter Securement and Careful Line Management During Positioning	Poorly secured IV lines are at risk for dislodgement or kinking, especially after major position changes or surgical manipulation. This can lead to complications.	• Use Tegaderm (3M Health Care, Minnesota, USA) with advanced adhesive for securement, visibility, and antiseptic coverage (Jenks et al.) [5] • Apply padding around catheter sites to reduce external pressure and prevent kinking/dislodgement
2) Regular Monitoring of IV Sites Intraoperatively and Standardized IV Assessment Protocols	Patent IVs can become dislodged or obstructed during position changes. Once sterile drapes are placed, anesthesia is solely responsible for IV monitoring. Literature supports standardized education and vigilance (Wallis et al., Marsh et al.) [6–8].	• Final inspection before draping • Saline flush after repositioning • Check straps/equipment for pressure on IV • Maintain visible IV access (when possible) or relocate high-risk infusions • Periodic palpation and inspection of limbs during surgery (when able to access)
3) Prompt Response to Signs of IV Infiltration	Delay in recognition and intervention can cause serious injuries (e.g., extravasation, compartment syndrome).	• Stop the infusion immediately • Elevate the affected limb • Administer antidotes per drug-specific protocol (if applicable)
4) Documentation and Communication	Intra-operative interdisciplinary communication helps ensure early recognition and prevention. Awareness across teams improves follow-up and patient safety (Tofani et al., Simin et al.) [9,10].	• Alert interdisciplinary team of any simple corrective issues (e.g., surgeon leaning on the line) • Communicate potential complications and postoperative concerns to all team members

In regard to catheter securement and line management during positioning, Jenks et al. [5] found using Tegaderm (3M Health Care, Minnesota, USA) with an advanced patented adhesive to be one of the most effective securement methods to decrease IV infiltration rates. This product, like many others utilized to secure IVs, provides stability during patient repositioning and offers some level of antiseptic protection. However, it is the advanced adhesive combined with transparency that makes this product an effective option for use in the operating room, as it allows for direct and continuous visualization of the catheter throughout surgery. Placing padding around the IV catheter site also helps to reduce localized external pressure on the line, which may serve to prevent catheter dislodgement or kinking during the procedure. When it comes to intraoperative assessments of IV sites, it is crucial that the anesthesia provider continue to reassess the IV for any complications throughout the procedure. Even initially patent IVs can become obstructed or displaced during positional adjustments, and overt external signs of injury are not always evident to the provider. While existing literature, including Wallis et al. and Marsh et al., suggests that nursing education on IV infiltration assessment and management can help reduce the incidence of infiltration injuries, there is limited evidence addressing how similar prevention can be achieved in the intraoperative setting [6-8]. Once surgery begins and the sterile drapes are in place, nursing staff typically no longer have access to visualize or assess peripheral IV sites. At that point, the responsibility for IV surveillance falls solely to the anesthesia team, making their vigilance essential for early detection and prevention of intraoperative IV infiltration injuries. There are; however, some recommended actions that would address the delayed detection of IV complications, including: final inspections of peripheral IV sites before draping or tucking hands for surgery, manual assessments of the IV site for continued flow and absence of resistance with a saline flush after any repositioning, reassessing for any straps or surgical equipment that may be exerting localized pressure on IV site once the patient is in final surgical positioning, ensuring visible access to the IV site (when possible) or relocating IV sites for infusions that may be high-risk for extravasation injury, and periodically palpating and inspecting limbs with an IV catheter throughout the surgery (if able to access). Prompt intervention for IV infiltration is essential to prevent serious injury to the patient. These management strategies include immediately stopping the infusion, elevating the affected limb to relieve pressure, and, depending on the infusion, administering antidotes in accordance with drug-specific protocols (if applicable) to avoid extravasation injury. Finally, interdisciplinary communication and documentation are important to allow for an organized response to the infiltration event. Tofani et al. and Simin et al. emphasize that open communication facilitates timely and sometimes simple corrective actions, such as alerting the surgeon to avoid leaning on a line to relieve external pressure [9,10]. Making all perioperative team members aware of potential complications allows for special considerations to be made for postoperative evaluation, monitoring, and follow-up.

Although clinical improvement was documented in this case with qualitative data, this case report is missing some details of recorded objective measurements to quantify the patient's improvement. Future reports on this topic should incorporate more objective metrics to quantify the severity of symptoms, such as limb circumference. This report focuses only on one patient and may not be widely generalizable; however, it emphasizes standardization of IV infiltration prevention strategies in the operative setting, as well as demonstrates the need for more studies to confirm effective intraoperative IV infiltration monitoring and management.

## Conclusions

This case demonstrates how easily intraoperative IV infiltration can go undetected when upper extremities are tucked for surgery and out of view of the anesthesia provider. In this case, the patient didn’t sustain long-term injuries secondary to the infiltrated IV, though the delayed recognition exposed him to possible avoidable risks, including compartment syndrome and neuropathy. While this patient was spared, this event does inspire a review of the guidelines and encourages the proposal of prevention and management strategies, specifically while in the operating room when dealing with obscured IV sites. There were found to be two main reasons for the increased difficulty of IV management during surgery: repositioning and tucking of the hands for surgery increase the chance of hidden infiltration, and there are no current standardized, evidence-based guidelines to assess or intervene with IV sites that are tucked for surgery. It is essential to develop clear protocols for routine limb checks and strategies to avoid infiltration while repositioning patients, while also developing clear intervention strategies in the event of IV infiltration to best avoid long-term injury. We recommend that anesthesia providers take the approach of taking a moment to inspect the IV site with every natural pause in surgery, such as when redosing antibiotics, to catch infiltration early on and minimize patient harm. Future studies may look at comparing manual routine inspections to technology-assisted monitoring of IV sites.
